# Comparative Analysis of 95 Patients with Different Severity in the Early Outbreak of COVID-19 in Wuhan, China

**DOI:** 10.1155/2020/4783062

**Published:** 2020-10-05

**Authors:** Fang He, Xue-feng Ding, Meng Cao, Hai-ying Gong, Xiang-zhen Fu, Jie Luo, Kui Liu, Zhou-zhou Tian, Lin Luo, Yu-yuan Fan, Ting Li, Qian-Jing Yao, Xiao-ju Chen, Xiang-lin Duan, Li Chen, Li Jiang

**Affiliations:** ^1^Department of Respiratory and Critical Care Medicine, Affiliated Hospital of North Sichuan Medical College, Nanchong, China; ^2^North Sichuan Medical College, Nanchong, China; ^3^Nanchong Central Hospital, The Second Clinical Medical College of North Sichuan Medical College, Nanchong, China; ^4^Department of Medical Intensive Care Unit, Affiliated Hospital of North Sichuan Medical College, Nanchong, China; ^5^Department of Infectious Disease, The First People's Hospital of Yibin, Yibin, China; ^6^Department of Cardiology, Shanghai Xuhui Central Hospital, Shanghai, China; ^7^Department of Radiology, Affiliated Hospital of North Sichuan Medical College, Nanchong, China; ^8^Department of Orthopedics, Wuhan Red Cross Hospital, Wuhan, China

## Abstract

**Objective:**

To explore the clinical characteristics of patients with different severity in the early outbreak of COVID-19, hoping to provide reference for clinical diagnosis and treatment.

**Methods:**

We retrospectively analyzed the clinical data of 95 COVID-19 patients in Wuhan Red Cross Hospital of China from January 17 to February 13, 2020. All patients were investigated with epidemiological questionnaires. Outcomes were followed up until April 1, 2020.

**Results:**

There were 53 males and 42 females, aged 22–84 years (mean 57.3 years). Clinical classification included 54 cases of common type, 27 cases of severe type, and 14 cases of critical type. Six patients had been exposed to the local Huanan seafood market. There were 38 clusters of COVID-19, including 27 family clusters and 11 work unit clusters. Common symptoms included fever (86 (90.5%) of 95), cough (73 (76.8%)), and fatigue (50 (52.6%)). Laboratory findings showed that the most common abnormalities were lymphopenia (75 (78.9%)), elevated D-dimer (60 (63.2%)), and elevated C-reactive protein (56 (58.9%)) on admission. All patients had abnormal chest computed tomography, showing patchy shadows or ground-glass opacities. Severe and critical cases were older, more likely to have shortness of breath, more likely to have underlying comorbidities, and more likely to have abnormal laboratory findings than common cases. The prognosis of patients with different degrees of severity was significantly different. All common and severe patients (100%) were cured and discharged from the hospital, while 10 (71.4%) of 14 critical patients died.

**Conclusions:**

COVID-19 has fast transmission speed and high pathogenicity. We must assess the severity of the disease and take corresponding treatment measures as early as possible.

## 1. Introduction

**Figure 1:**
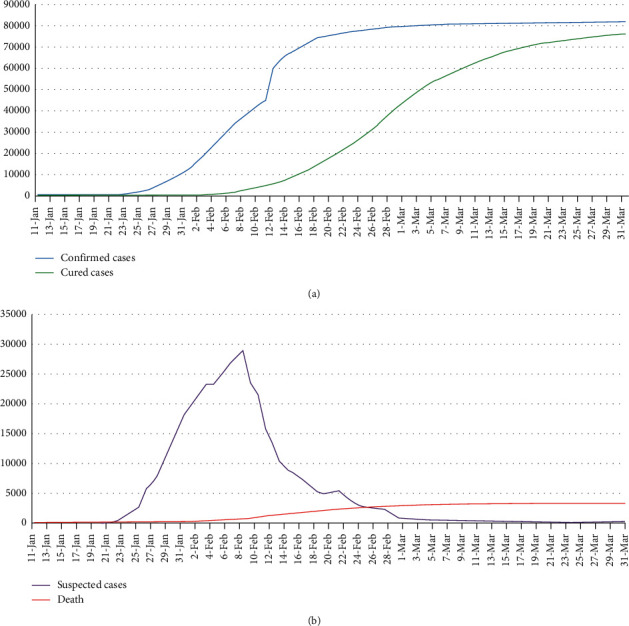
(a) Confirmed cases and cured cases and (b) suspected cases and deaths of novel coronavirus pneumonia (NCP) from January 11 to March 31, 2020, in mainland China. The data obtained from the official website of the National Health Commission of the People's Republic of China (http://www.nhc.gov.cn/xcs/yqtb/list_gzbd.shtml).

Since December 2019, a cluster of unknown pneumonia cases with exposure to the local Huanan seafood wholesale market has been reported in Wuhan, Hubei Province, China [1]. A novel coronavirus was isolated from the airway epithelial cells of infected patients through laboratory etiological detection, and the pathogen of this unknown viral pneumonia was preliminarily determined to be a novel coronavirus [2, 3]. The virus was provisionally named 2019 novel coronavirus (2019-nCoV) by the World Health Organization (WHO) on January 12 and was subsequently named coronavirus disease 2019 (COVID-19) on February 11, 2020 [1]. The International Committee on Taxonomy of Viruses (ICTV) termed it severe acute respiratory syndrome coronavirus 2 (SARS-CoV-2) as it is very similar to the one that caused the SARS outbreak (SARS-CoVs). SARS-CoV-2 is spreading widely around the world, and the number of confirmed cases and deaths is rising. The WHO characterized COVID-19 as a pandemic on March 11, 2020. As of March 31 (24:00 Beijing Time), 2020, there have been 81,554 confirmed cases of COVID-19 in mainland China, including 3,312 deaths [4]. Figures1(a) and 1(b)show the dynamic data report of the SARS-CoV-2 epidemic in mainland China from January 11 to March 31, 2020.

To date, some researchers have retrospectively analyzed the data of COVID-19 patients, but the cases are relatively limited, and the situation of patients may be different in different regions and different periods. In this study, we comprehensively explore the epidemiological and clinical characteristics of 95 COVID-19 patients admitted to Wuhan Red Cross Hospital in the early stage of the outbreak, hoping to provide reference for the diagnosis and treatment of this disease.

## 2. Methods

### 2.1. Study Design and Participants

This study was approved by the Institutional Ethics Committee of Wuhan Red Cross Hospital (No. 2020008). Wuhan Red Cross Hospital is one of the first batches of COVID-19 designated diagnosis and treatment hospitals in Wuhan, China. The 95 COVID-19 patients hospitalized in Wuhan Red Cross Hospital of China from January 17 to February 13, 2020, were enrolled in this study.

### 2.2. Inclusion Criteria

All patients enrolled in this study were diagnosed according to “Guidelines for the Diagnosis and Treatment of Novel Coronavirus (2019-nCoV) Infection (Trial Version 5)” by the National Health Commission of the People's Republic of China [5]. Patients who met the following criteria were confirmed cases: SARS-CoV-2 nucleic acid testing was positive in the upper respiratory tract via nasopharyngeal or oropharyngeal swab samples, or lower respiratory tract via expectorated sputum samples, endotracheal aspirate samples, or bronchoalveolar lavage samples.

### 2.3. Exclusion Criteria

There was no exposure history of confirmed or suspected cases, and SARS-CoV-2 nucleic acid tests and serum SARS-CoV-2 specific IgM and IgG antibodies were negative for multiple times in the course of the disease, and chest computed tomography (CT) showed no signs of pneumonia for many times.

### 2.4. Clinical Classification

According to the clinical and imaging manifestations, the confirmed COVID-19 patients were divided into common type, severe type, and critical type. Common type patients had fever, respiratory tract symptoms, and other symptoms, and imaging showed signs of pneumonia. Severe type adult patients met any of the following criteria: (1) shortness of breath, respiratory frequency ≥30 times/min; (2) oxygen saturation ≤93% in the resting state; and (3) partial arterial oxygen pressure (PaO_2_)/fraction of inspiration O_2_ (FiO_2_) ≤ 300 mmHg. Pulmonary imaging showed that patients with lesions that progress significantly more than 50% within 24–48 hours can also be diagnosed as severe type. Critical type patients met one of the following conditions: (1) respiratory failure requiring mechanical ventilation; (2) shock; (3) critical organ failure requiring intensive care unit (ICU) care.

### 2.5. Data Collection

The epidemiological and symptom data were obtained through questionnaire survey of patients. Professionals carried out epidemiological case investigations on patients one by one in wards. The written informed consent from all patients involved was obtained before enrolment. We obtained clinical, laboratory, radiology, treatment, and outcome data from patients' medical records. The recorded information included demographic data, symptoms, signs, exposure history, underlying comorbidities, laboratory test results, chest radiology, complications, treatment, and outcome data. All data were reviewed by Jiang L, MD. Clinical outcomes were followed up until April 1, 2020. The date of disease onset was defined as the day when the symptoms were noticed. The time from onset of symptoms to isolation, first medical assistance, admission, diagnosis, and the time from admission to ICU, discharge, and death were recorded. We compared the clinical characteristics and hospitalization time of different clinical types such as common, severe, and critical patients.

### 2.6. Statistical Analysis

Continuous variables were described by mean, median, and interquartile range (IQR) values. Categorical variables were described as frequency and percentages in each category. Means for continuous variables were compared by one-way ANOVA when the data were normally distributed; otherwise, the Kruskal–Wallis H test was used. The chi-square test, Fisher's test, linear-by-linear association, or Goodman–Kruskal gamma was used for comparison between categorical variables according to different data characteristics and analysis purposes. A *P* value of less than 0.05 (*P* < 0.05) was considered statistically significant. All statistical analysis was performed with the use of SPSS software, version 26.0.

## 3. Results

### 3.1. Epidemiological Characteristics

There were 53 males (55.8%) and 42 females (44.2%), with a male-to-female ratio of 1.26 : 1.00. The age ranged from 22 to 84 years, with a mean age of 57.3 years and a median age of 60 years. The average age of men was 57.9 years, slightly higher than that of women (56.5 years). Clinical classification included 54 cases of common type, 27 cases of severe type, and 14 cases of critical type. The age of the common type group was significantly lower than that of the severe and critical type groups, but there was no significant difference between the severe and critical types. Age was significantly different among different clinical types and positively correlated with the severity of the disease. Most patients (61.1%) were exposed to suspected or confirmed cases. Of the 95 patients, 6 (6.3%) had been exposed to the local Huanan seafood market, and 27 (28.4%) had been exposed to the local agricultural market, most of whom were consumers. There were 38 clusters of SARS-CoV-2 infection, including 27 family clusters and 11 work unit clusters. Hospital staffs and their families were infected with 2 cases, respectively (Table 1).

### 3.2. Clinical Characteristics

The most common symptom was fever, and the body temperature was mostly between 38.1 and 39.0 degrees celsius. Common symptoms included fever (86 (90.5%) of 95), cough (73 (76.8%)), fatigue (50 (52.6%)), shortness of breath (44 (46.3%)), chest tightness (41 (43.2%)), and dyspnea (32 (33.7%)). Other symptoms included chill (24 (25.3%)), myalgia (22 (23.2%)), diarrhea (21 (22.1%)), anorexia (17 (17.9%)), pharyngalgia (17 (17.9%)), expectoration (17 (17.9%)), headache (16 (16.8%)), nausea (16 (16.8%)), joint pain (15 (15.8%)), chest pain (11 (11.6%)), nasal obstruction (9 (9.5%)), vomiting (7 (7.4%)), rhinorrhea (6 (6.3%)), stomachache (2 (2.1%)), and conjunctival congestion (2 (2.1%)). Of the 95 patients, 50 (52.6%) had one or more underlying comorbidities. Hypertension (33 (34.7%)), cardiovascular disease (13 (13.7%)), diabetes (8 (8.4%)), chronic obstructive pulmonary disease (6 (6.3%)), and malignancy (3 (3.2%)) were the common underlying comorbidities (Table 1).

### 3.3. Laboratory Findings

Laboratory tests such as leucocyte count, neutrophil count, platelet, hemoglobin, creatinine kinase, creatine kinase-MB, blood urea nitrogen, creatinine, potassium, and calcium were usually within the normal range. The most frequent abnormality was lymphopenia, including 75 cases (78.9%) on primary admission and 89 cases (93.7%) during hospitalization. Other common abnormalities included elevated C-reactive protein (56 (58.9%) of 95), elevated D-dimer (60 (63.2%) of 95), and elevated lactate dehydrogenase (38 (56.7%] of 67). On admission, 23 patients (23/95) had prolonged prothrombin time (>13.5 s), 14 patients (14/18) had elevated erythrocyte sedimentation rate (>20 mm/h), 9 (9/82) had elevated creatine kinase (>200 u/l), 3 (3/33) had elevated creatine kinase-MB (>24 u/l), and 3 (3/15) had elevated cardiac troponin I (>0.04 *µ*g/l). Liver enzymes elevated in 36 patients (37.9%) on admission, including 32 cases of aspartate aminotransferase (AST) elevation and 25 cases of alanine aminotransferase (ALT) elevation. Leukocyte count, neutrophil count, C-reactive protein, urea nitrogen, creatinine, lactate dehydrogenase (LDH), albumin, calcium, prothrombin time, and D-dimer were significantly different among common, severe, and critical patients (*P* < 0.05), indicating that the more severe the disease, the more prone it is to abnormal laboratory indexes (Table 2).

Most patients were examined for respiratory pathogens, including *Mycoplasma pneumoniae*, *Chlamydia pneumoniae*, influenza A and B, parainfluenza, avian influenza, respiratory syncytial virus, and adenovirus, 2 of whom were complicated with influenza virus infection, while the rest were not infected with other viruses.

We tested the nasopharyngeal or oropharyngeal swabs of 95 patients by the real-time reverse transcription-polymerase chain reaction (RT-PCR) assay and found that the SARS-CoV-2 nucleic acid test was positive in 62 patients and negative in 33 patients on admission. However, after repeated SARS-COV-2 nucleic acid tests, 95 patients were eventually confirmed as confirmed cases because they all had SARS-CoV-2 nucleic acid positive results. Serum SARS-CoV-2 specific IgM and IgG antibodies were tested in all patients who were cured and discharged from the hospital, and the follow-up results confirmed that they were all positive for IgM or IgG antibodies.

### 3.4. Radiological Features

All patients had chest CT or X-ray abnormalities, including 78 cases of bilateral lung lesions and 17 cases of unilateral lesions. Chest CT scan showed patchy shadows or ground-glass opacities (GGO) in all patients' lungs on admission, of which 9 cases were accompanied by grid shadows. Chest CT showed GGO in 73 cases (76.8%), patchy shadows in 31 cases (32.6%), paving stone sign in 24 cases (25.3%), increased lymph node counts in 24 cases, lymphadenectasis in 15 cases, pleural effusion in 7 cases, and pericardial effusion in 3 cases on admission. Chest CT reexamination during hospitalization showed lymph node counts increased in 37 cases, lymphadenectasis in 17 cases, pleural effusion in 16 cases, and pericardial effusion in 5 cases. In addition, 49 patients showed lesions progression, including 25 cases of common type, 18 cases of severe type, and 6 cases of critical type.

**Figure 2:**
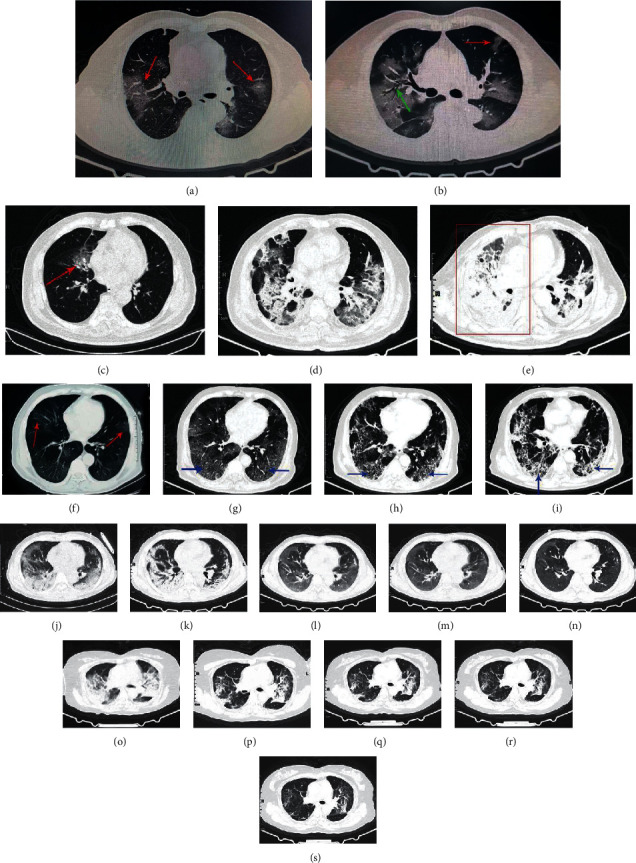
Chest computed tomographic images of COVID-19 patients. (a) and (b) show typical ground-glass opacities (marked by red arrow) and patchy shadows in both lungs of COVID-19 patients. The green arrow shows bronchial inflation sign. Case 1: (c), (d), and (e) are chest CT images of a 75-year-old dead man at the time of onset, the 14th day, and the 21st day after onset, respectively. The patient's lung lesions became more and more serious, and the right lung finally presented a “white lung” like change (as shown in red rectangle). Case 2: (f), (g), (h), and (i) are chest CT images of a 69-year-old severe male patient on admission, the 7th, 14th , and 21st days, respectively. The lesions showed fibrotic progression. The part pointed by the blue arrow shows “paving stone sign.” Case 3: (j), (k), (l), (m), and (n) are chest CT images of a 60-year-old severe male patient on admission, the 6th, 12th, 18th, and 24th days, respectively. The patient's lung lesions gradually improved after comprehensive treatment. Case 4: (o), (p), (q), (r), and (s) are chest CT images of a 58-year-old woman on admission, the 6th, 12th, 18th, and 26th days, respectively. The patient's bilateral lung lesions gradually improved after symptomatic treatment.

Imaging manifestations of COVID-19 patients were mostly subpleural patchy shadows, segmental or subsegmental ground-glass opacities in the early stage, often accompanied by thickening of blood vessels. In the advanced stage, the lesions increased in number and scope and developed into ground-glass opacities and consolidation shadows with multileaf involvement of both lungs. Bronchial inflation sign and “crazy-paving pattern” could be found. Severe patients showed diffuse lesions of both lungs and even “white lung” changes. Pulmonary lesions were improved, and fibrous lesions were formed in convalescence. Pulmonary hilar and mediastinal lymphadenectasis and a large amount of pleural effusion were rare.  Figure 2 shows the chest CT images of several cases of COVID-19.

Common complications of COVID-19 patients included acute respiratory distress syndrome (ARDS) (16 (16.8%)), followed by secondary bacterial pneumonia (14 (14.7%)), respiratory failure (8 (8.4%)), and gastrointestinal bleeding (8 (8.4%)). Statistical analysis showed that severe and critical patients were more prone to complications, especially in critical patients (Table 3). There were significant differences among common, severe, and critical patients (*P* < 0.05).

### 3.5. Treatment

All patients were treated with oseltamivir and Lianhua Qingwen granules (LHQWG, a Chinese traditional patent medicine) before hospital admission. Patients took one bag of Lianhua Qingwen granules (specification: 6 g per bag) three times a day. Of the 95 patients, 85 patients (89.5%) received moxifloxacin before hospital admission. Antibacterial therapy after admission included cephalosporin antibiotics (20 (21.1%)), piperacillin sodium tazobactam sodium (20 (21.1%)), levofloxacin (14 (14.7%)), etimicin (7 (7.4%)), meropenem (6 (6.3%)), and linezolid (5 (5.3%)). Antiviral therapy after admission included oseltamivir (31 (32.6%)), lopinavir plus ritonavir (55 (57.9%)), abidol (41 (43.2%)), ganciclovir (33 (34.7%)), ribavirin (24 (25.3%)), interferon-alpha (13 (13.7%)), and acyclovir (4 (4.2%)). Interferon-alpha was given by atomizing inhalation at a dose of 5 million units or 50 mg per dose twice a day for 14 d. Glucocorticoid therapy included methylprednisolone (29 (30.5%)) and dexamethasone (3 (3.2%)). A total of 95 patients (100%) received respiratory support therapy, including 65 cases of low flow nasal catheter oxygen inhalation, 13 cases of high-flow nasal cannula oxygen therapy, 12 cases of noninvasive ventilation, and 5 cases of invasive ventilation. Other treatments included gamma globulin (10 (10.5%)) and immunoglobulin (9 (9.5%)).

Of the 95 patients, 85 (89.5%) were isolated before hospital admission, and the median time from onset of symptoms to isolation was 1.0 day. The median time from onset of symptoms to first medical assistance, diagnosis, and hospital admission was 2.0 days (IQR, 0–7.0), 8.0 days (IQR, 3.5–12.0), and 11.0 days (IQR, 7.0–13.0), respectively. Of the 95 patients, 18 (18.9%) were admitted and transferred to the ICU because of the development of organ dysfunction. The median time from hospital admission to the ICU, discharge, and death was 6.0 days (IQR, 2.0–10.0), 19.0 days (IQR, 14.0–29.5), and 18.0 days (IQR, 11.0–25.0), respectively (Table 4).

### 3.6. Outcome

As of April 1, 2020, a total of 85 patients (89.5%) had been discharged, and 10 patients (10.5%) had died.

## 4. Discussion

The recent emergence of COVID-19 puts the world on alert. At present, it is urgent to update the understanding of the diseases caused by SARS-CoV-2 infection. Thus, we conduct the current study aiming to help health workers recognize and understand this disease. This is a descriptive study on the epidemiology and clinical characteristics of the COVID-19 patients. We report here a cohort of 95 patients in the early stage of the outbreak of the COVID-19 epidemic. We have made a comprehensive analysis of patients with different severity levels. Our findings provide important parameters for further analyses, including clinical, laboratory, radiology, treatment, and outcome data.

In this study, we observe a greater number of men than women in the 95 cases of COVID-19, which is similar to severe acute respiratory syndrome coronavirus (SARS-CoV) [6] and Middle East respiratory syndrome coronavirus (MERS-CoV) [7] and consistent with recent reports [8–11]. It is considered that female's reduced susceptibility to viral infection could be attributed to the protection from X chromosome and sex hormones [12]. Females can develop enhanced innate and adaptive immune responses than males and are less susceptible to many infections of bacterial, viral, parasitic, and fungal origin.

Similar to SARS-CoV and MERS-CoV infections, COVID-19 patients showed symptoms of viral pneumonia, including fever, cough, fatigue, and bilateral lung infiltration in the most cases [13]. Besides, more recent reports have reported gastrointestinal symptoms and asymptomatic infections, especially among young children [14, 15]. There is likely a lot of variability in the clinical presentation, including asymptomatic or mild cases that may never present to healthcare services. In addition, there is a concern that asymptomatic and mild patients may more likely spread the virus due to not seeking medical assistance in time. Therefore, early identification and prevention of transmission is of paramount significance.

We found that the underlying health of the COVID-19 patients likely plays a critical role in overall susceptibility. In our study, male patients with underlying comorbidities were more likely to be infected with SARS-CoV-2, and the condition was relatively serious due to the weaker immune functions. Therefore, early identification, early diagnosis, and timely treatment of such cases are of great importance. It is necessary to keep alert to these vulnerable patients following SARS-CoV-2 infection.

Consistent with recent literature reports, our cohort also observed that the most common laboratory abnormalities in COVID-19 patients were lymphopenia, elevated C-reactive protein, and elevated D-dimer. Most patients had obvious lymphopenia during hospitalization. Severe patients and critical patients were more likely to have a progressive lymphopenia. Lymphocytes damage might be an important factor leading to exacerbations of the patients [16]. It is recommended to use immunoglobulin to enhance the immunity of severe patients. In our cohort, some patients with progressive lymphopenia used gamma globulin or immunoglobulin, which showed a good curative effect. In addition, we found that leukocyte elevation, neutrophil elevation, and C-reactive protein elevation were more common in severe and critical patients, which indicated that severe and critical patients may be more prone to secondary bacterial infection. Severe and critical patients were more prone to a variety of abnormal laboratory results such as prothrombin time, D-dimer, urea nitrogen, creatinine, and albumin, which indicated that critical patients might be more prone to multiple organ dysfunction.

In our study, the SARS-CoV-2 nucleic acid test of some patients before definite diagnosis was still negative for multiple times, but epidemiology, chest CT, blood routine, and other exclusion tests indicated that SARS-CoV-2 infection was highly likely. Therefore, the early identification of COVID-19 should be performed taking into account the patient's epidemiology, clinical manifestations, radiology, laboratory findings, respiratory pathogen detection and so on. Chest CT images of the COVID-19 patients have high specificity and diagnostic value. Understanding CT evolution characteristics of COVID-19 patients can provide important basis for early prevention and control, early diagnosis, and treatment of the disease. In addition, chest CT can be used to assess the severity of lung involvement in COVID-19.

In terms of treatment, as most patients were treated with moxifloxacin before admission, the antibacterial treatment rate of the patients was on the high side, which was not in conformity with the medical standards. In fact, antibacterial therapy cannot improve the overall prognosis of patients and may even lead to the imbalance of flora to some extent. Besides, there were no significant therapeutic differences among different antiviral regimens. Since our study is a retrospective study, more randomized controlled studies are needed to clarify this in the future. In China, traditional Chinese medicine is widely used to treat COVID-19 [17]. It is reported that more than 85% of COVID-19 patients were treated with traditional Chinese medicine [18]. In our study, all patients were treated with Lianhua Qingwen granules (LHQWG, a Chinese patent medicine). LHQWG was first used in the treatment of influenza, and studies have found that it is superior to oseltamivir in improving the symptoms of influenza A virus infection [19]. LHQWG was recommended for clinical treatment of patients with common symptoms according to the fifth edition of COVID-19's diagnosis and treatment guideline issued by the China Health Commission [5] due to its remarkable efficacy in fighting influenza. A study has suggested that routine treatment combined with LHQWG may improve clinical symptoms including fever, cough, sputum, fatigue, and dyspnea, suggesting that LHQWG may be an effective treatment for COVID-19 patients [20]. There was, however, no significant difference in the improvement rate of clinical symptoms in this study. The mechanism of its action needs to be further studied and clarified.

To date, there is no effective therapeutics for COVID-19. The existing treatment methods included antiviral, antibacterial, symptomatic, and supportive treatment. The therapies with plasma and antibody obtained from convalescent patients have been proposed as a treatment method for severe and critical patients [5], and some critical patients had remarkable effects. So far, many anti-novel coronavirus drugs and vaccines have been developed, but none has been widely used in clinical practice yet. We believe that these problems will eventually be solved with the deepening of research.

In the early stage of the outbreak of the COVID-19 epidemic, due to the haste of time and preparation, there is a lack of experience in the diagnosis and treatment of the disease. Our study is a real-world single-center cohort study. Our cohort showed that patients with different severity groups had certain differences in age, gender, symptoms, complications, laboratory test indexes, treatment, outcomes, etc. Our study provides important reference for the diagnosis and treatment of COVID-19 infection, as well as certain experience and lessons for the subsequent epidemic prevention, treatment, and health system construction. We will further summarize our experience and continuously improve it in the follow-up research.

Since the end of 2019, the outbreak of COVID-19 has been drawing tremendous attention around the world. World governments and researchers have taken swift measures to control the outbreak and conduct the etiological studies. There are still many uncertainties regarding the virus-host interaction and the evolution of the epidemic, especially when the epidemic will reach its peak. The COVID-19 epidemic in China has been initially controlled, but the overseas epidemic situation is not optimistic. COVID-19 has extensive transmission and strong pathogenicity, and human beings are generally susceptible. As there is no effective therapeutics or vaccines, the best way to deal with COVID-19 is to control the source of infection, early diagnosis, report, isolation, supportive treatment, and timely publish epidemic information to avoid unnecessary anxiety and panic.

We must draw lessons from SARS-CoV and MERS-CoV incidents. Therefore, we must make early assessment of the severity of the disease, judge and predict the development of novel coronavirus pneumonia, and take corresponding measures in time.

## Figures and Tables

**Table 1:** Baseline characteristics of COVID-19 patients.

Characteristics	All patients	Disease severity			*P* value
		Common	Severe	Critical	
Number	95	54 (56.8%)	27 (28.4%)	14 (14.7%)	—
Age, yrs					
Mean ± SD	57.3 ± 14.7	51.7 ± 14.1	63.7 ± 12.5	66.4 ± 11.2	＜0.001
Median (IQR)	60 (48.0–67.0)	52.0 (41.5–64.0)	65.0 (52.0–73.0)	66.5 (59.0–76.0)	—
Age groups, *n* (%)					＜0.001
18–40 yrs	15 (15.8)	13 (24.1)	2 (7.4)	0 (0)	—
41–65 yrs	52 (54.7)	33 (61.1)	12 (44.4)	7 (50.0)	—
≥66 yrs	28 (29.5)	8 (14.8)	13 (48.1)	7 (50.0)	—
Sex, *n* (%)					
Male	53 (55.8)	22 (40.7)	20 (74.1)	11 (78.6)	0.003
Female	42 (44.2)	32 (59.3)	7 (25.9)	3 (21.4)	—
Medical staffs and their families, *n* (%)	4 (4.2)	2 (3.7)	1 (3.7)	1 (7.1)	0.839
Exposure history, *n* (%)	33 (34.7)	16 (29.6)	12 (44.4)	5 (35.7)	0.417
Exposure to Huanan seafood market	6 (6.3)	2 (3.7)	3 (11.1)	1 (7.1)	0.430
Exposure to agricultural market	27 (28.4)	14 (25.9)	9 (33.3)	4 (28.6)	0.784
Infection clusters, *n* (%)	38 (40.0)	23 (42.6)	13 (48.1)	2 (14.3)	0.093
Contacts with suspected or confirmed patients, *n* (%)	58 (61.1)	34 (63.0)	16 (59.3)	8 (57.1)	0.901
Comorbidities, *n* (%)					
Hypertension	33 (34.7)	14 (25.9)	14 (51.9)	5 (35.7)	0.069
Diabetes	8 (8.4)	2 (3.7)	4 (14.8)	2 (14.3)	0.164
Cardiovascular and cerebrovascular diseases	13 (13.7)	3 (5.6)	6 (22.2)	4 (28.6)	0.026
Symptoms and signs, *n* (%)					
Fever	86 (90.5)	48 (88.9)	25 (92.6)	13 (92.9)	0.822
Maximum temperature, °C	—	—	—	—	0.294
<37.3	9 (9.5)	6 (11.1)	2 (7.4)	1 (7.1)	—
37.3–38.0	18 (18.9)	13 (24.1)	3 (11.1)	2 (14.3)	—
38.1–39.0	49 (51.6)	29 (53.7)	13 (48.1)	7 (50.0)	—
>39.0	19 (20.0)	6 (11.1)	9 (33.3)	4 (28.6)	—
Cough	73 (76.8)	41 (75.9)	19 (70.4)	13 (92.9)	0.262
Fatigue	50 (52.6)	23 (42.6)	17 (63.0)	10 (71.4)	0.070
Shortness of breath	44 (46.3)	16 (29.6)	16 (59.3)	12 (85.7)	＜0.001
Chest tightness	41 (43.2)	19 (35.2)	17 (63.0)	5 (35.7)	0.049
Dyspnea	32 (33.7)	14 (25.9)	11 (40.7)	7 (50.0)	0.155
Chill	24 (25.3)	13 (24.1)	7 (25.9)	4 (28.6)	0.938
Myalgia	22 (23.2)	10 (18.5)	11 (40.7)	1 (7.1)	0.025
Diarrhea	21 (22.1)	15 (27.8)	3 (11.1)	3 (21.4)	0.234
Anorexia	17 (17.9)	10 (18.5)	5 (18.5)	2 (14.3)	0.930
Pharyngalgia	17 (17.9)	12 (22.2)	3 (11.1)	2 (14.3)	0.436
Expectoration	17 (17.9)	9 (16.7)	3 (11.1)	5 (35.7)	0.140
Headache	16 (16.8)	8 (14.8)	7 (25.9)	1 (7.1)	0.261
Nausea	16 (16.8)	10 (18.5)	4 (14.8)	2 (14.3)	0.881
Joint pain	15 (15.8)	8 (14.8)	6 (22.2)	1 (7.1)	0.435
Chest pain	11 (11.6)	4 (7.4)	5 (18.5)	2 (14.3)	0.319
Nasal obstruction	9 (9.5)	5 (9.3)	2 (7.4)	2 (14.3)	0.773
Rhinorrhea	6 (6.3)	3 (5.6)	2 (7.4)	1 (7.1)	0.940
Vomiting	7 (7.4)	4 (7.4)	3 (11.1)	0 (0)	0.434
Conjunctival congestion	2 (2.1)	2 (3.7)	0 (0)	0 (0)	0.460
Stomach ache	2 (2.1)	0 (0)	1 (3.7)	1 (7.1)	0.200
Isolation, *n* (%)	85 (89.5)	49 (90.7)	24 (88.9)	12 (85.7)	0.856
Days from onset to first visit	2.0 (0–7.0)	2.0 (0–5.8)	4.5 (1.0–7.3)	2.0 (0–6.0)	0.303
Days from onset to diagnosis	8.0 (3.5–12.0)	7.0 (3.0–12.0)	8.5 (4.0–12.3)	9.0 (5.8–13.3)	0.564
Days from first visit to diagnosis	4.0 (0–8.0)	3.0 (0–8.0)	2.5 (0–6.5)	6.0 (0.5–9.5)	0.558
Days from onset to isolation	1.0 (0–5.0)	1.0 (0–4.5)	2.0 (0–6.0)	0 (0–2.0)	0.222
Days from onset to hospitalization	11 (7–13)	10 (6–12)	11 (7–15)	11 (6.0–13.5)	0.350

Data are presented as *n* (%), mean ± standard deviation (SD), or medians (interquartile ranges, IQR).

**Table 2:** Laboratory findings of COVID-19 patients on hospital admission.

Laboratory findings	All patients	Disease severity			*P* value
		Common	Severe	Critical	
Number	95	54 (56.8)	27 (28.4)	14 (14.7)	—
Blood routine					
Leukocyte count, ×10⁹/L	5.6 (4.5–7.0)	5.0 (4.3–6.7)	5.8 (4.9–7.4)	7.1 (5.5–10.3)	0.010
<4	17 (17.9)	11 (20.4)	5 (18.5)	1 (7.1)	0.513
4–10	71 (74.7)	42 (77.8)	20 (74.1)	9 (64.3)	0.583
>10	7 (7.4)	1 (1.9)	2 (7.4)	4 (28.6)	0.003
Neutrophil count, ×10⁹/L	4.0 (2.8–5.1)	3.6 (2.6–4.4)	4.5 (3.3–5.8)	5.0 (4.1–9.3)	0.002
<1.5	5 (5.3)	5 (9.3)	0 (0)	0 (0)	0.135
1.5–7	79 (83.2)	46 (85.2)	23 (85.2)	10 (71.4)	0.446
>7	11 (11.6)	3 (5.6)	4 (14.8)	4 (28.6)	0.046
Lymphocyte count, ×10⁹/L	1.0 (0.8–1.4)	1.1 (0.8–1.6)	1.0 (0.6–1.3)	0.9 (0.5–1.2)	0.137
<1.5	75 (78.9)	40 (74.1)	23 (85.2)	12 (85.7)	0.409
Platelet count, × 10⁹/L	168 (126–220)	166.5 (126–226.3)	185 (125–227)	158 (117–206)	0.780
<150	36 (37.9)	20 (37.0)	9 (33.3)	7 (50.0)	0.569
Hemoglobin, g/L	136 (125–147)	133 (125.8–148)	137 (118–145)	140.5 (129–147)	0.755
C-reactive protein, mg/L	32.6 (7.6–80.1)	12.9 (3.1–44.3)	52.6 (27.2–112.6)	80.1 (22.0–118.5)	＜0.001
>10	56 (58.9)	26 (48.1)	21 (77.8)	9 (64.3)	0.035
Blood biochemistry					
Aspartate aminotransferase, U/L	34.4 (22.8–46.0)	29.2 (19.7–40.6)	37.7 (27.5–46.0)	41.2 (19.3–60.7)	0.108
>40	32 (33.7)	14 (25.9)	10 (37.0)	8 (57.1)	0.080
Alanine aminotransferase, U/L	27.6 (17.8–42.2)	24.5 (13.2–36.1)	30.0 (18.4–45.0)	35.2 (24.4–46.0)	0.172
>40	25 (26.3)	11 (20.4)	9 (33.3)	5 (35.7)	0.315
Urea nitrogen, mmol/L	4.0 (2.8–5.1)	3.5 (2.7–4.5)	5.1 (3.1–9.1)	4.4 (3.6–6.5)	0.004
>7.5	15 (15.8)	3 (5.6)	9 (33.3)	3 (21.4)	0.004
Creatinine, umol/L	65.8 (51.6–81.8)	63.5 (49.3–77.0)	72.1 (54.0–105.0)	67.4 (59.4–80.3)	0.011
>133	3 (3.2)	0 (0)	3 (11.1)	0 (0)	0.020
LDH, U/L	264.4 (192.1–372.2)	245.3 (179.6–329.7)	301.3 (199.8–402.9)	388.0 (311.4–561.4)	0.010
Creatine kinase, U/L	56.9 (41.1–98.7)	52.0 (36.5–89.8)	56.2 (46.9–136.6)	73.8 (50.9–359.2)	0.210
Albumin, g/L	35.8 (32.0–39.6)	37.2 (34.4–39.8)	34.8 (30.3–39.6)	29.6 (28.1–33.8)	＜0.001
Potassium, mmol/L	3.7 (3.3–4.0)	3.6 (3.4–3.9)	3.8 (3.3–4.2)	3.6 (3.0–3.9)	0.201
Calcium, mmol/L	2.2 (2.2–2.3)	2.3 (2.2–2.4)	2.2 (2.1–2.3)	2.2 (2.1–2.3)	0.039
Coagulation function					
Prothrombin time, s	12.9 (12.2–13.5)	12.6 (12.1–13.1)	13.8 (12.1–14.3)	13.4 (12.7–14.2)	0.002
>13.5	23 (24.2)	4 (7.4)	15 (55.6)	4 (28.6)	＜0.001
D-dimer, mg/L	0.71 (0.36–3.39)	0.51 (0.31–0.84)	1.90 (0.68–23.81)	2.60 (0.59–21.46)	＜0.001
>0.5	60 (63.2)	26 (48.1)	22 (81.5)	12 (85.7)	0.002
First nucleic acid test					0.323
Positive	62 (65.3)	32 (59.3)	19 (70.4)	11 (78.6)	—
Negative	33 (34.7)	22 (40.7)	8 (29.6)	3 (21.4)	—

Data are presented as *n* (%) or medians (interquartile ranges, IQR). LDH, lactate dehydrogenase.

**Table 3:** Complications of COVID-19 patients.

Complications	All patients	Disease severity			*P* value
		Common	Severe	Critical	
Number	95	54 (56.8)	27 (28.4)	14 (14.7)	—
Complication	20 (21.5)	2 (3.7)	7 (25.9)	11 (78.6)	＜0.001
Secondary bacterial pneumonia	14 (14.7)	1 (1.9)	7 (25.9)	6 (42.9)	＜0.001
ARDS	16 (16.8)	1 (1.9)	8 (29.6)	7 (50.0)	＜0.001
Acute kidney injury	6 (6.3)	0 (0)	0 (0)	6 (42.9)	＜0.001
Acute liver injury	4 (4.2)	0 (0)	0 (0)	4 (28.6)	＜0.001
Gastrointestinal bleeding	8 (8.4)	2 (3.7)	2 (7.4)	4 (28.6)	0.011
Respiratory failure	8 (8.4)	0 (0)	0 (0)	8 (57.1)	＜0.001
Hypoproteinemia	5 (5.3)	0 (0)	0 (0)	5 (35.7)	＜0.001
Septic shock	5 (5.3)	0 (0)	0 (0)	5 (35.7)	＜0.001
Metabolic acidosis	4 (4.2)	0 (0)	0 (0)	4 (28.6)	＜0.001
Cardiac insufficiency	4 (4.2)	0 (0)	0 (0)	4 (28.6)	＜0.001
Myocarditis	3 (3.2)	0 (0)	0 (0)	3 (21.4)	＜0.001
Coagulopathy	2 (2.1)	0 (0)	0 (0)	2 (14.3)	0.003
Mental symptoms	2 (2.1)	0 (0)	1 (3.7)	1 (7.1)	0.200
Hypoxic encephalopathy	2 (2.1)	1 (1.9)	0 (0)	1 (7.1)	0.313

Data are presented as *n* (%). ARDS, acute respiratory distress syndrome.

**Table 4:** Treatments and outcomes of COVID-19 patients.

Treatments and outcomes	All patients	Disease severity			*P* value
		Common	Severe	Critical	
Number	95	54 (56.8)	27 (28.4)	14 (14.7)	NA
Treatment					
HFNC	13 (13.7)	0 (0)	6 (22.2)	7 (50.0)	＜0.001
NIV	12 (12.6)	0 (0)	5 (18.5)	7 (50.0)	＜0.001
IV	5 (5.3)	0 (0)	0 (0)	5 (35.7)	＜0.001
Antiviral therapy	91 (95.8)	52 (96.3)	25 (92.6)	14 (100.0)	0.513
Abidol	41 (43.2)	20 (37.0)	14 (51.9)	7 (50.0)	0.382
Oseltamivir	31 (32.6)	18 (33.3)	7 (25.9)	6 (42.9)	0.541
Ganciclovir	33 (34.7)	17 (31.5)	10 (37.0)	6 (42.9)	0.697
Lopinavir plus ritonavir	55 (57.9)	32 (59.3)	15 (55.6)	8 (57.1)	0.949
Ribavirin	24 (25.3)	12 (22.2)	7 (25.9)	5 (35.7)	0.583
Interferon	14 (14.7)	5 (9.3)	5 (18.5)	4 (28.6)	0.155
Moxifloxacin^∗^	85 (89.5)	47 (87.0)	24 (88.9)	14 (100)	0.368
Levofloxacin	14 (14.7)	6 (11.1)	6 (22.2)	2 (14.3)	0.412
Linezolid	5 (5.3)	1 (1.9)	1 (3.7)	3 (21.4)	0.013
Piperacillin	20 (21.1)	8 (14.8)	10 (37.0)	2 (14.3)	0.055
Meropenem	6 (6.3)	0 (0)	4 (14.8)	2 (14.3)	0.015
Fluconazole	3 (3.2)	0 (0)	1 (3.7)	2 (14.3)	0.024
Glucocorticoids	32 (33.7)	11 (20.4)	12 (44.4)	9 (64.3)	0.003
Immunoglobulin	19 (20.0)	12 (22.2)	4 (14.8)	3 (21.4)	0.727
ICU admission	18 (18.9)	2 (3.7)	7 (25.9)	9 (64.3)	＜0.001
Days from hospital admission to ICU	6 (2–10)	NA	4 (1–8)	7.0 (3.0–11.5)	0.272
Outcome					
Died	10 (10.5)	0 (0)	0 (0)	10 (71.4)	＜0.001
Improved and discharged	85 (89.5)	54 (100.0)	27 (100)	4 (28.6)	＜0.001
Days from hospitalization to discharge	19 (14.0–29.5)	16.5 (13.0–21.8）	28 (18–41)	NA	＜0.001
Days from hospitalization to death	18 (11–25)	NA	NA	18 (11–25)	NA

Data are presented as *n* (%) or medians (interquartile ranges, IQR). NA: not appropriate; HFNC, high-flow nasal cannula oxygen therapy; NIV, noninvasive ventilation; IV, invasive ventilation. ∗The usage of moxifloxacin in this table is the data of COVID-19 patients before admission.

## Data Availability

There are no linked research data sets for this submission. The raw/processed data required to reproduce these findings cannot be shared at this time as the data also form part of an ongoing study.

## Abbreviations

2019-nCoV: 2019 novel coronavirus
WHO: World Health Organization
COVID-19: Corona virus disease 2019
ICTV: International Committee on Taxonomy of Viruses
SARS-CoV-2: Severe acute respiratory syndrome coronavirus 2
CT: Computed tomography
PaO_2_: Partial arterial oxygen pressure
FiO_2_: Fraction of inspiration O_2_
ICU: Intensive care unit
IQR: Interquartile range
SD: Standard deviation
AST: Aspartate aminotransferase
ALT: Alanine aminotransferase
LDH: Lactate dehydrogenase
RT-PCR: Real-time reverse transcription-polymerase chain reaction
GGO: Ground-glass opacities
ARDS: Acute respiratory distress syndrome
LHQWG: Lianhua Qingwen granules
HFNC: High-flow nasal cannula oxygen therapy
NIV: Noninvasive ventilation
IV: Invasive ventilation
SARS-CoV: Severe acute respiratory syndrome coronavirus
MERS-CoV: Middle East respiratory syndrome coronavirus.
